# Traditional Chinese medicine injections: where we are after 80-year development

**DOI:** 10.1186/s13020-022-00681-w

**Published:** 2022-11-08

**Authors:** Wenwen Zheng, Yiyang Wu, Hanlu Gao, Defang Ouyang

**Affiliations:** 1 Department of Clinical Laboratory, The Sixth Affiliated Hospital of Sun Yat-Sen University, Guangzhou, China; 2 State Key Laboratory of Quality Research in Chinese Medicine, Institute of Chinese Medical Sciences (ICMS), University of Macau, Macau, China

**Keywords:** Traditional Chinese medicine injections, Safety concern, Effectiveness, Pharmacoeconomics, Re-evaluation, Quality control

## Abstract

Traditional Chinese medicine (TCM) injection is the combination of modern pharmaceutical technology and traditional Chinese prescription, which was born in 1941 and played a great role in the backward medical conditions at that time. However, the debate over TCM injections has never stopped due to adverse drug reactions (ADRs). The regulation on TCM injections has been further strengthened since 2017, which has prompted many TCM injections to carry out re-evaluations on quality, safety, efficiency as well as pharmacoeconomics, which made significant changes and progress. This review presented an up-to-date analysis of the types, amounts, and ADRs of TCM injections based on the published data and literature. This review also summarized the potential reasons for the ADRs and re-evaluation strategies. This review will provide some useful clues for TCM injections and their clinical use.

## Background

Traditional Chinese medicine (TCM) injection is defined as a sterile preparation (solution, powder, concentrated solution, emulsions, etc.) intended for injecting into the human body, which is extracted from traditional Chinese herbs [1]. TCM injection is the combination of TCM theories and modern pharmaceutical technology. Compared with the traditional dosage forms (e.g. pill, powder, paste, etc.) of TCM, TCM injection has the characteristic of rapid effect, which can be used in acute and severe diseases in TCM treatments. In clinical application, TCM injections are mainly used for fighting cancer, promoting blood circulation, heat-clearing and detoxifying [2].

Unlike other TCM dosage forms that have evolved over thousands of years, TCM injection has been developed for about 80 years (Table 1). Traces of TCM injections in China can date back to the Second World War. During that period, China faced a situation where medical care was extremely lacked, and the clinical curative effect brought by traditional TCM dosage forms was also very limited. TCM injections were researched during that urgent situation. In 1941, the successful development of the Chaihu injection marked the birth of TCM injection, which saved lots of lives. In December 1954, Chaihu injection was industrialized in Wuhan Pharmaceutical Factory and became the first industrialized product of TCM injections in China. During the 1960s–1970s, TCM injections entered an era of rapid development. According to statistics, the number of TCM injection products reached 1400 in the 1980s [3]. After the 1980s, regulators began to draw up a series of regulations and drug standards to strengthen TCM injection management. However, due to the very limited research about TCM injections in the early stages, more and more safety issues of TCM injections have been exposed. Many serious adverse drug reactions (ADRs) caused by TCM injections, such as Yuxingcao injection and Ciwujia injection, have raised a lot of safety concerns about TCM injections [4]. After that, the state drug regulator issued a series of policies and regulations successively to promote the quality of TCM injections, emphasizing the rigorous approval and re-evaluation of injections [5–7]. In 2022, the Lianbizhi injection became the first delisted TCM injection for the reason of post-marketing evaluation failure [8].

**Table 1:** The brief history of TCM injections

Year	Events	Description
1941	Chaihu injection was successfully developed	Chaihu injection marked the birth of TCM injections
1954	Wuhan Pharmaceutical Factory put the Chaihu injection into production	The first industrially produced TCM injection in China
1960s–1980s	TCM injections increased sharply	TCM injection products reached up to 1400 in the 1980s
1990	Shuanghuanglian powder injection was developed and produced by industrialization	The first TCM powder injection
2006	Yuxingcao injection was urgently stopped in the clinic	Yuxingcao injection caused 2282 ADRs from 2006 to 2008, and 22 people died [14]
2007	Released *Basic technical requirements for traditional Chinese medicine and natural medicine injections*	Requirements in terms of safety, effectiveness and necessity
2009	Released *Notice on Carrying out the Safety Re-evaluation of Traditional Chinese Medicine Injections*	The first time to propose the re-evaluation of TCM injections
2010	Proposed seven technical principles to standardize and guide the safety re-evaluation of TCM injections	These principles involve evaluation of non-clinical research, clinical research, production process, quality control, enterprise’s ability to risk control, benefits and risks, risk management
2017	Released *Opinions on Deepening the Reform of the Review and Approval System and Encouraging the Innovation of Drugs and Medical Devices*	Strict the review and approval of injections; Carry out the re-evaluation of injections
2020	Some TCM injections were recommended by Diagnosis and Treatment Protocol to treat pneumonia	TCM injections were mainly used to treat severe and critical illness
2022	Lianbizhi injection was delisted from the TCM market	The first TCM injection to be delisted due to re-evaluation failure

Regulations and policies released are in italics

For a long time, the adverse drug reaction (ADR) reports caused by TCM injections have resulted in hot debates on TCM injections, and also raised safety concerns among people. In recent years, especially after 2017, the strengthening supervision of TCM injections has brought changes to TCM injections. This review aims to give a summary of the changes in TCM injections from the current situation, ADRs, and re-evaluation, hoping to provide the basis for re-evaluating TCM injections and their clinical use.

## Current situation of TCM injection

**Fig. 1:**
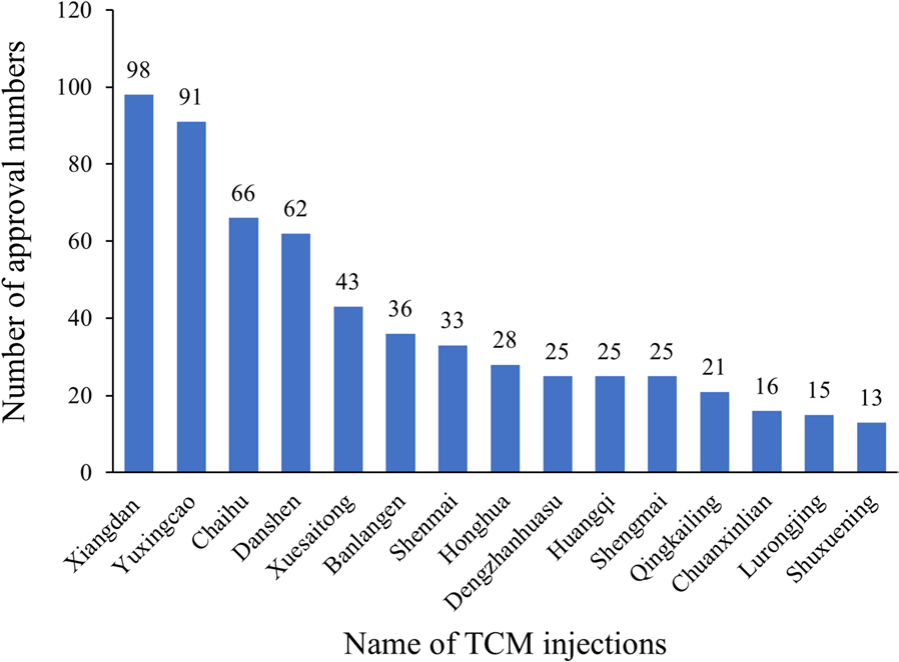
Number of approval numbers for top 15 TCM injections

TCM injections are special preparations with the approval number “Z”, which means that their raw materials are Chinese herbs. On the other hand, those with the approval number “H” belong to the category of chemical drugs. Up to July 6, 2022, the data from the National Medical Products Administration (NMPA) showed that China had approved 128 TCM injections, involving 872 approval numbers and 196 manufacturing enterprises [9]. The top 15 approval numbers of TCM injections are shown in Fig. 1. Among approved TCM injections, 8 kinds of TCM injections were covered by the latest *Drug Lists of National Essential Medicine (version 2018)* [10]. In the latest *Medicines List for National Basic Medical Insurance (version 2021)*, 47 TCM injection items with 44 unique serial numbers are contained and almost all of them are restricted to be used by second-class or above hospitals [11]. TCM injections have also been used in treatments during the COVID-19 pandemic. In the 4th trial version of *Diagnosis and Treatment Protocol for COVID-19,* four kinds of TCM injections have been recommended to treat pneumonia in China for the first time, and up to eight kinds in the latest 9th version to treat severe or critically ill patients [12, 13].

## Adverse drug reactions caused by TCM injections

TCM is rooted in the clinical practice experience for thousands of years. It is usually taken through oral administration, thus some unwanted harmful substances can be eliminated by the physiological barriers and the liver first-pass effect. However, the parenteral injection of herb extractions will bypass these barriers and enter the human body directly, which although takes effect rapidly but greatly increases the risk of side effects [15]. With the wide application of TCM injections in clinical practice, a lengthening list of adverse effects has been reported. The harmful impurities in TCM injections, such as the biological macromolecules, endotoxin, and pyrogen, are generally considered to cause ADRs. It has been found that the ADRs caused by TCM injections have the characteristics of multiple, universality, diversity and uncertainty [16]. The main ADR types of TCM injection appearing in clinical use are pyrogen reaction, allergic reaction, and anaphylactoid reaction. Among them, the anaphylactoid reaction has the highest incidence rate [17].

**Fig. 2:**
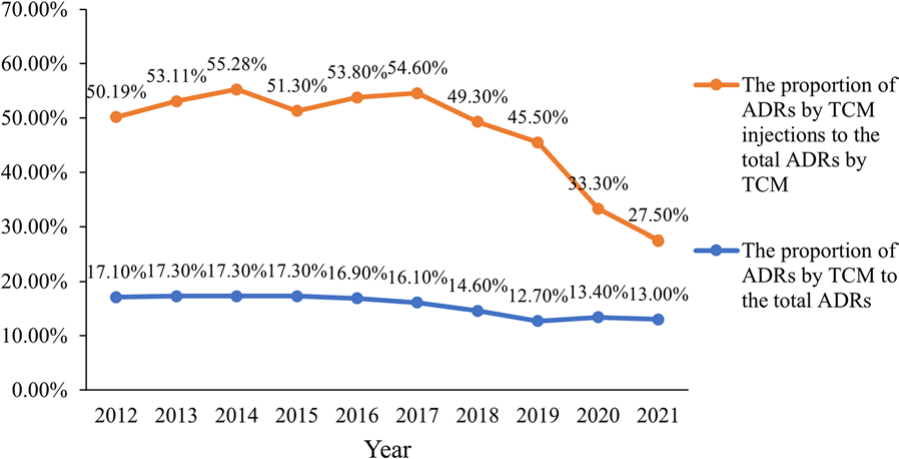
Trends in the number of ADR reports by TCM injections from 2012 to 2021

Figure 2 shows the statistic for the ADR reports on TCM injections in the past 10 years. In the past 10 years, the proportion of ADR reports caused by TCM to the total ADR reports is stable, while the proportion of ADR reports caused by TCM injections has changed greatly. From 2012 to 2017, ADR reports caused by TCM injections account for more than 50% of all ADR reports related to TCM. But after 2017, the proportion of TCM injection ADRs dropped significantly year by year and accounted for 27.5% of the TCM ADRs in 2021, which may be relative to the strict regulation and restricted clinic use after 2017 [7].

### Factors influencing adverse drug reaction

#### The ingredients of TCM injections are complex but poorly studied

Different from chemical injections with well-defined ingredients, TCM injections are made from herbal extracts with complex compositions, which are very difficult to carry out research with total active components. Moreover, due to the low-level scientific research in the early period, most TCM injections only did ambiguous research on their active ingredients and harmful impurities. Thus, the safety and effectiveness of TCM injections can’t be guaranteed [18].

The pharmacokinetic study is an important method to reveal the effect and the in vivo process of active ingredients. The pharmacokinetic studies of TCM injections mainly involve the interaction within the components and the interaction studies in combination with chemical drugs. Considering the complex composition of most TCM injections and their unknown pharmacodynamic material basis, the pharmacokinetic research of TCM injections is much more difficult than that of chemical drugs. At present, the pharmacokinetic studies of TCM injections are not insufficient, especially in the human body, which leads to uncontrollable risks in clinical use [19].

#### The quality of raw materials in traditional Chinese medicine is uneven

The occurrence of ADR is mainly related to the lack of rigorous management of raw materials. TCMs are extracted from herbs, which are influenced by environmental factors, origin, agriculture, collecting, harvesting, and primary processing of raw materials [20]. The differences in the growing areas and harvesting dates have a direct impact on the effective components of traditional Chinese herbs. For example, *Fritillaria* growing in Zhejiang has different therapeutic outcomes from those growing in Sichuan, although they belong to the same genus [21]. Besides, the phenomenon of confusing the names of traditional Chinese herbs also contributes to the uneven quality of raw materials [22].

#### Crude preparation process and quality control problems

The quality control system plays a pivotal role in drug quality. The preparation processes of TCM injections determine the safety and effectiveness of the products. Compared with chemical injections, the preparation processes of TCM injections are more complicated, including extraction, filtration, precipitation, purification, sterilization, etc., which greatly increases the difficulties of quality control [23]. Furthermore, in the early years, the preparation processes of most TCM injections were simple and not refined, which made quality control even harder. For example, the quality standards of the same TCM injection may have a big difference between different companies, and even the quality of injections by the same company may vary from batch to batch [24]. For example, a previous study tested the quality of Shuanghuanglian injections by 12 different companies and found that these injections had different content of chlorogenic acid [25]. At present, some TCM injections are still limited to using behindhand traditional preparation processes. These traditional methods have many drawbacks including a large loss of effective ingredients, insufficient removal of harmful substances, and mutual reactions between ingredients by improper process design.

#### Improper clinical use

Besides the manufacturing and quality problems of TCM injections, another issue is the irrational use of TCM injections in clinical practice. Firstly, improper use may attribute to non-standard drug instructions. As the important basis for rational drug use, the drug instruction approved by the drug administration department should contain detailed information such as drug ingredients, clinical usage and precautions [26]. However, very little information is provided in the TCM injection instructions, which provides very limited clinical guidance. Secondly, the doctors may lack knowledge of TCM. TCM injections are still based on TCM theory. Therefore, it is necessary to follow the dialectical treatment of traditional Chinese medicine theories in clinical usage. Inappropriate use of TCM injections may not only have poor therapeutic effects but also lead to adverse drug reactions [27, 28]. In addition, other clinical factors should also be mentioned, such as the selection of solvent, speed of administration, compatibility of medicines, route of administration, and dosage, which also may cause ADRs [29–31].

## Re-evaluation of TCM injections

The early approval threshold for TCM injections was low, and most TCM injections had no thorough research on safety and efficacy [18]. In recent years, TCM injections have been questioned a lot because of frequent ADRs. Due to serious adverse events, the government must pay more attention to the quality problems of TCM injections. According to the *Opinions on Deepening the Reform of the Review and Approval System and Encouraging the Innovation of Drugs and Medical Devices* released in 2017, for the purpose of evaluating both chemical and TCM injections in safety, efficacy, and quality controllability, enterprises must conduct the comprehensive research at approval, and the post-marketing researches, including the active ingredients, mechanism of action, and clinical efficacy [7].

Recently the re-evaluation of TCM injections has achieved certain positive results. For example, the ADR proportion caused by TCM injections has decreased continually since the rigorous requirements for the injections in approval and re-valuation were proposed in 2017 (Fig. 2). Moreover, up to July 11, 2022, the Announcements issued by NMPA revised the contents of instruction manuals for 31 kinds of TCM injections [32]. These fully affirm the validity and necessity of re-evaluation. The enterprises should evaluate and improve the quality of TCM injection products as soon as possible following the relative regulations and policies (Table 2).

**Table 2:** Comparisons between the TCM injection regulation status and the requirements for general injections

	Requirements for general injections	TCM injection regulation status
Pre-clinical research	Definitive studies of all components in the injection, including the structures and properties of components and interactions between components	• The complexity of Chinese herbal medicines makes it difficult to identify the active ingredients and the harmful substances • Some TCM injections have already met the requirements of *689 Principle* on the material basis
Clinical research	Large-scale and high-quality clinical trials are required to demonstrate the safety and efficacy of the injections	• Did not undergo rigorous clinical trials when TCM injections were approved • Most TCM injections do not have the ability to carry out eligible clinical trials to demonstrate their clinical safety and efficacy
Preparation process and quality control	• For chemical injection, the production processes are relatively simple and controllable • Modern manufacture with advanced technologies	• The production processes are more complicated, making quality control very difficult • Outdated preparation methods such as the alcohol-water method are still adopted by some TCM injections on the market
	• Specific quality control will be used for each well-defined ingredient in the injections • The indicators usually specify the upper and lower limits of the component contents	• Low-level quality control standards and methods • Limited control is used for complicated TCM injections, only a few components are tested for quality control • It is hard to set standards to measure unknown ingredients in TCM injections
Post-market regulation	• Submit information on side effects and quality management to the authorities regularly • Some injections will require Phase IV clinical trials	• Seriously inadequate post-market supervision • All TCM injections need to take the re-evaluation

### Demonstrate safety and effectiveness with scientific evidence

Efficacy is the key to a drug. Usually, a drug must be evaluated in strict scientific clinical trials with hundreds to thousands of patients. However, due to the low level of research and limited technical conditions at the early stage, most TCM injections did not undergo rigorous clinical trials before approval [33]. Up to July 6, 2022, only 21 kinds of TCM injections among 128 approved TCM injections had relevant clinical trial information in the *ClinicalTrials.gov* database [34].

In clinical practice, TCM injections are mostly used in combination with other chemical drugs. For instance, among 4382 cases associated with the Shuanghuanglian injection in the clinic, 82.79% of injections were used in combination with antibiotic drugs [35]. It is quite necessary to use reliable and scientific evidence to evaluate the efficacy of TCM injections being used alone [36]. If the real efficacy of TCM injections is not clear, it is hard to distinguish their real contribution to combination therapies.

Identifying the material basis of TCM injections is a prerequisite for evaluating clinical efficacy and safety. In 2006, the *Basic Technical Requirements for Traditional Chinese Medicine and Natural Medicine Injection* made requirements on the ingredients of TCM injection [5]: for injections made of active ingredients, the content of a single ingredient should be no less than 90%; for multi-component TCM injections, the content of well-defined components in the total solids should be no less than 60% and the measurable components should be no less than 80% of total solids; raw materials, intermediates and preparations should be studied to establish fingerprints respectively, and the correlation study between them should be carried out; the components whose structure is clarified should be reflected in the fingerprint, generally not less than 90% of clarified components. These requirements are collectively called *689 Principle*. A previous review summarized that some TCM injections have satisfied the requirements of 689 Principle, some even with higher standards [37]. For example, a company claimed on its website that they have completed a study of the material basis of Tanreqing injection: among the total solids of Tanreqing injection, the structures of over 80% of components are well-defined, over 80% of components are measurable, and 93% of the well-defined components can be identified in the fingerprints [38].

Pharmacokinetic studies investigate drug action mechanisms and in vivo processes, which can provide scientific guidance for clinical uses. In recent years, some pre-clinical and clinical pharmacokinetic studies have been carried out for the re-evaluation of TCM injections. However, most of them focused on pre-clinical research, while only a few studies related to clinical studies [19]. It is well-known that the clinical pharmacokinetic study in the human body is of great significance for the safety and effectiveness of a drug in clinical uses. Thus, more large-scale and standardized clinical trials of TCM injections should be carried out in the future.

Evidence-based medicine is another powerful method for evaluating the efficacy of TCM injections. A number of TCM injection studies related to randomized control trials (RCTs) and meta-analysis were published every year [39–41]. Danhong injection (DHI) is a kind of multi-target drug. To evaluate the efficacy and safety of DHI to treat symptomatic chronic stable angina, a rigorously designed randomized controlled trial was conducted in 920 patients with moderate symptomatic stable angina. The results showed that after 14-day of DHI use, the angina episodes were significantly reduced and without any additional risk. The follow-up visit found that the health condition specific to angina was improved for at least 90 days. This study further identified the anti-angina therapeutic modules of DHI on the effective population by developing a systemic modular approach. This approach will be helpful to facilitate the modernization of Chinese medicine in confirming therapeutic effects and revealing therapeutic mechanisms [40]. Moreover, the professional TCM clinical evidence database (EVDS) which has been established in 2016 can act as a very powerful tool to provide a reference for the TCM injection clinical effectiveness evaluation [42].

### Strengthen the quality management of raw herb materials

As one of the dosage forms with the highest quality requirements, TCM injection is required higher standards of consistency and controllability than other traditional Chinese medicine products. As the important raw materials for TCM injection production, the quality of medicinal plant raw materials should be controlled first.

In 2002, the first *Quality Management Standards of Chinese Herbal Medicine* was released to control the quality of herb medicine by using Good Agricultural Practice (GAP) [43]. GAP is the basic criterion to standardize the production and management of Chinese herbs, and regulates the processes of herbs such as collection, storage, and processing. As of February 2016, 167 kinds of Chinese herbal medicines from 129 companies have passed the GAP certification [44]. But this GAP was canceled in 2016 because it played a limited role in the continual management. In 2022, the new GAP certification was released to further promote the standardized production of Chinese herbal medicine [45]. Compared with the old version of GAP, the newly released GAP further refines the whole process of quality management in Chinese herbs. Enterprises should strengthen their management of the quality of raw materials from the herb source by new GAP standards.

### Modernize the preparation processes and improve the quality control

Most enterprises still adopt outdated methods and low-standard quality control. To ensure high-standard and controllable production processes, enterprises should speed up their transformation to more advanced and scientific production processes. In 2016, the NMPA clarified the requirements for enterprises to achieve drug traceability [46]. The quality traceability system refers to recording the drug information from manufacture to market to quickly and effectively trace any quality issues. A good traceability system is very important to the quality management of TCM injections. For example, although the Xiyanping injection has completed a large number of non-clinical safety and pharmacology studies and post-marketing clinical safety studies from 2009 to 2013 [47], this TCM injection was still urgently recalled for causing severe adverse reactions such as chills and fever in 2017 [48]. This gives us enlightenment that a one-time evaluation cannot ensure the life-long safety of a drug, and safety evaluation should be a systematic project that runs through the entire life cycle of TCM injections.

### Pharmacoeconomics evaluation of TCM injections

The purpose of the pharmacoeconomic evaluation is to rationally select and use drugs to achieve efficient, safe, and economical use of limited medical and health resources to get the optimal treatment effect and the minimal economic burden. TCM injections occupy a huge share of the TCM market compared with other dosage forms of TCMs, accounting for one-third of the total TCM sales in hospitals [49]. while the therapeutic effects of TCM injections in clinical use do not match the high investment. For example, one study showed that the cost of Shuanghuanglian injection and Qingkailing injection was nearly 10 to 30 times higher than that of oral dosage forms when obtaining the same therapeutic effect [50]. In order to effectively control unnecessary drug costs, on the one hand, the clinical cost and effectiveness of commonly used TCM injections in *National Basic Medical Insurance* should be investigated to compare with other alternative dosage forms. On the other hand, the cost of ADR treatment from TCM injections should be monitored too.

## Conclusion

Current paper summarized and discussed the brief history, current situation, ADRs, and re-evaluation and progress of TCM injections. Due to the limited research on TCM injections at the early stage, there was insufficient evidence in nearly all aspects of clinical efficiency, safety, quality control, manufacturing process, and pharmacoeconomics to support the clinical usage of most TCM injections. TCM injections are currently at a critical moment to do the post-marketing evaluation. Although the qualities of some TCM injections have been improved in recent years, re-evaluation of TCM injections needs to be further promoted to obtain more acceptance and recognition. For instance, the quality management of raw herbs should be strengthened; convincible studies of material basis and pharmacokinetics should be finished; the modernization of preparation processes should be accelerated; the necessity for using TCM injections should be assessed through pharmacoeconomic evaluation. In general, the post-market evaluation has played a positive role in the quality control of TCM injections. Under the increasingly strict requirement of the government and market, the quality control and clinic use of TCM injections will be more standardized.

## Data Availability

Not applicable.

## Abbreviations

TCM: Traditional Chinese medicine
TCMs: Traditional Chinese medicines
ADR: Adverse drug reaction
ADRs: Adverse drug reactions
NMPA: The National Medical Products Administration
RCTs: Randomized control trials
GAP: Good Agriculture Practice
DHI: Danhong injection
EVDS: The Traditional Chinese Medicine Clinical Evidence Database System
